# A rapid and accurate methylation‐sensitive high‐resolution melting analysis assay for the diagnosis of Prader Willi and Angelman patients

**DOI:** 10.1002/mgg3.637

**Published:** 2019-04-29

**Authors:** Igor Ribeiro Ferreira, Wilton Darleans dos Santos Cunha, Leonardo Henrique Ferreira Gomes, Hiago Azevedo Cintra, Letícia Lopes Cabral Guimarães Fonseca, Elenice Ferreira Bastos, Juan Clinton Llerena, Zilton Farias Meira de Vasconcelos, Letícia da Cunha Guida

**Affiliations:** ^1^ Laboratório de Alta Complexidade Instituto Nacional da Saúde da Mulher da Criança e do Adolescente Fernandes Figueira Fiocruz Rio de Janeiro Brazil; ^2^ Departamento de Genética Instituto Nacional da Saúde da Mulher da Criança e do Adolescente Fernandes Figueira Fiocruz Rio de Janeiro Brazil

**Keywords:** Angelman syndrome, high‐resolution melting, MS‐HRM, MS‐PCR, Prader Willi syndrome

## Abstract

**Background:**

Prader Willi (PWS) and Angelman (AS) syndromes are rare genetic disorders characterized by deletions, uniparental disomy, and imprinting defects at chromosome 15. The loss of function of specific genes caused by genetic alterations in paternal allele causes PWS while the absence in maternal allele results AS. The laboratory diagnosis of PWS and AS is complex and demands molecular biology and cytogenetics techniques to identify the genetic mechanism related to the development of the disease. The DNA methylation analysis in chromosome 15 at the *SNURF*‐*SNRPN* locus through MS‐PCR confirms the diagnosis and distinguishes between PWS and AS. Our study aimed to establish the MS‐PCR technique associated with High‐Resolution Melting (MS‐HRM) in PWS and AS diagnostic with a single pair of primers.

**Methods:**

We collected blood samples from 43 suspected patients to a cytogenetic and methylation analysis. The extracted DNA was treated with bisulfite to perform comparative methylation analysis.

**Results:**

MS‐HRM and MS‐PCR agreed in 100% of cases, identifying 19(44%) PWS, 3(7%) AS, and 21(49%) Normal. FISH analysis detected four cases of PWS caused by deletions in chromosome 15.

**Conclusion:**

The MS‐HRM showed good performance with a unique pair of primers, dispensing electrophoresis gel analysis, offering a quick and reproducible diagnostic.

## 1 INTRODUCTION

Prader Willi (PWS) (Cassidy & Schwartz, 1998) and Angelman (AS) (Cassidy & Schwartz, 1998) syndromes are rare genetic diseases with an estimated prevalence of 1/15.000 live births affecting both sexes equally (Cassidy & Driscoll, 2009). Although PWS and AS syndromes occur in the same region in the genome, they present very distinct clinical characteristics. PWS is the most frequent cause of secondary obesity characterized by severe neonatal hypotonia, dysmorphic facial features, early onset of hyperphagia, development of morbid obesity, short stature, hypogonadism, learning difficulties, behavioral and cognitive impairment; on the other hand, AS is characterized by severe developmental disabilities, seizures, speech deficits, motor spasticity, and epilepsy (Clayton‐Smith & Laan, 2003; Ramsden, Clayton‐Smith, Birch, & Buiting, 2010).

PWS and AS syndromes are associated with chromosomal abnormalities in the majority of cases. Abnormalities are occurring at the paternal allele on chromosome 15q11‐q13 results in PWS (Sun et al., 1996), while abnormalities in the maternal copy of the same region cause AS. The genetic mechanisms leading to PWS include (1) deletion of the syndrome‐associated region (~75%), (2) uniparental disomy (UPD) (~25%), or (3) imprinting center defect (IC) (~1% of PWS) (Smith & Hung, 2017). The frequency of rearrangements occurs in ~5% of the individuals and can be balanced or unbalanced (Kuslich, Kobori, Mohapatra, Gregorio‐King, & Donlon, 1999; Reeve et al., 1993; Robinson et al., 1993; Smith et al., 1991; Yip, 2014). These genetic and epigenetic alterations occur in a highly complex region in 15q11‐q13 with a series of imprinted and nonimprinted genes.

The laboratory diagnosis of PWS and AS is a challenge and demands several molecular and cytogenetic methods to elucidate the genetic mechanism that leads to the development of the syndrome (Cassidy & Driscoll, 2009). DNA methylation analysis is a robust approach for the diagnosis of PWS due to the capacity to diagnose correctly the main imprinting error caused by all three genetic mechanisms (Deletions, UPD, and IC defects) (Ramsden et al., 2010). The *SNURF‐SNRPN locus* [MIM# 176270], contains a CpG island with a variable methylation pattern according to the origin of the allele, and most of the CpG dinucleotides in this region are extensively methylated in maternal chromosome 15, but not methylated on paternal chromosome 15 (Buller et al., 2000). Amplification of GC‐rich sequences is essential for screening and diagnostic of some genetic diseases. At the molecular level, the paternal and maternal copies of this region can be distinguished by DNA methylation analysis of the *SNURF‐SNRPN* gene, being capable of detecting PWS or AS syndromes (“Diagnostic testing for Prader‐Willi and Angelman syndromes,” 1996; Dos Santos, Mota, Rocha, & Ferreira de Lima, 2016; White, Durston, Harvey, & Cross, 2006).

Even though DNA sequencing is considered the “gold standard” for mutation screening, this methodology remains relatively expensive, laborious, and time‐consuming. Many other methods for mutation screening have been developed to track changes in an individual's DNA. These techniques include analysis of single‐strand conformational polymorphisms (SSCP) (Orita, Iwahana, Kanazawa, Hayashi, & Sekiya, 1989), denaturing gradient gel electrophoresis (DGGE) (Lerman & Silverstein, 1987), denaturing high performance liquid chromatography and (Xiao & Oefner, 2001) temperature gradient capillary electrophoresis (GCEC) (Li, Liu, Monroe, & Culiat, 2002). All of these methods require postprocessing steps, which include a separation of the sample into a gel or other matrix. Fluorescently labeled probe methods such as dual hybridization probes (Wittwer, Herrmann, Moss, & Rasmussen, 1997), exonuclease (TaqMan) (Heid, Stevens, Livak, & Williams, 1996), or hairpin (Molecular Beacons) (Tyagi & Kramer, 1996) may be used for mutation detection. However, probes would be specific to DNA sequence with a priori knowledge. Therefore, these methods are not susceptible to mutational scanning, since mutational scanning requires methods that can detect mutations in larger regions. Also, some of the above methods are not automated and laborious while others are complex, expensive and require specialized instrumentation.

High resolution melting (HRM) technique is a simple method based on PCR. In the presence of saturating concentrations of DNA‐binding dyes, the specific sequence of the amplicon determines the melting behavior as the solution temperature is increased. The intensity of fluorescence decreases as the double‐stranded DNA becomes single‐stranded and the dye is released. The melt temperature (Tm) in which 50% of the DNA is in the double‐stranded state can be approximated by taking the derivative of the melting curve. The characteristic melting curve can be used to detect variations in the DNA sequence in the amplicon without the need for any post‐PCR processing. The method is easy to use, highly sensitive, specific, low cost and produces rapid turnaround in the sample (De Leeneer, Coene, Poppe, De Paepe, & Claes, 2008; Kramer et al., 2009), making HRM an attractive option for the detection of mutational variants associated with diseases with applications in clinical diagnostic laboratories.

Also, HRM is a nondestructive method. Therefore, subsequent analysis of the sample by other techniques, such as gel electrophoresis or DNA sequencing, can still be performed after HRM analysis. These features make HRM ideal for use in routine diagnostic settings. Due to its numerous advantages, MS‐HRM methodology has been widely applied in diagnostic laboratories for the screening of mutations associated with diseases. Since it was first introduced for genotyping in 2003 (Wittwer, Reed, Gundry, Vandersteen, & Pryor, 2003), articles have been published applying the HRM technique to detect mutations in a wide range of genes such as EGFR (Takano et al., 2008), BRAF (Pichler et al., 2009), and BRCA (van der Stoep et al., 2009). For further elucidation about DNA pattern methylation, bisulfite conversion and DNA sequencing is a method of choice, because it provides detailed information on the methylation pattern of individual DNA molecules at single CG site resolution. (Dos Santos et al., 2016; Kosaki, McGinniss, Veraksa, McGinnis, & Jones, 1997; Zeschnigk, Lich, Buiting, Doerfler, & Horsthemke, 1997).

This study aimed to establish the Methylation‐Sensitive High‐Resolution Melting (MS‐HRM) with a previous DNA bisulfite treatment to diagnose PWS, and AS. MS‐HRM allows detection of differences up to 0.2°C showing the capacity to distinguishing both syndromes with a single pair of primers (Mehta, Daniel, & McNevin, 2017).

## 2 MATERIALS AND METHODS

### 2.1 Sample collection and DNA isolation

Our institutional review board approved this study under the number 45767015.0.0000.5269. Blood samples were collected from 43 individuals, with clinical criteria for Prader–Willi Syndrome. The patients were enrolled in Fernandes Figuera National Institute (IFF/FIOCRUZ). As a control, blood samples from healthy individuals were collected. DNA extraction was performed using the DNAeasy Blood & Tissue Kit obtained from Qiagen (Valencia, CA) following instructions provided by the standard kit protocol. The DNA quality was assessed with NanoDrop Spectrophotometer. The DNA purity was also evaluated through the wavelength 260/280 and 260/230, avoiding contaminants.

### 2.2 Cytogenetic and FISH analysis

The cytogenetic study was performed from peripheral blood sample stimulated by phytohemagglutinin according to standard protocol. The analysis was carried out by Karyotype G banding (GTG) (450/550 bands per haploid set) according to ISCN 2016. The FISH analysis was performed in nucleus and metaphases chromosomal preparations according to standard protocols using SNRPN/GABRB3 probe (Cytocell Inc).

### 2.3 Bisulfite DNA treatment

The genomic DNA input was 250 ng/μl to be modified with sodium bisulfite using the EZ DNA^™^ methylation kit (Zymo Research, USA). The final protocol step was to elute in 10 μl of nuclease‐free water according to the manufacturer's instructions. The modified DNA was quantified and was assessed with NanoDrop Spectrophotometer.

### 2.4 Methylation‐specific PCR

The Methylation‐Specific PCR (MS‐PCR) was performed in 25 μl of a mixture containing 10 pmol of each primer (ThermoFisher Scientific, USA), 12.5 μl Maxima^®^ Hot Start PCR Master Mix (ThermoFisher Scientific, USA) with Hot Start Taq DNA polymerase and nuclease‐free water. Primer‐Blast online tool (Ye et al., 2012) was used to design primers *SNURF‐SNRPN* locus (GenBank accession number: NG_009157.1) for methylated and nonmethylated alleles: 5′‐Ggatttttgtattgcggtaaataag ‐3′ (forward/PWS_F), 5′‐Caactaaccttacccactccatc‐3′ (reverse/PWS_R). As a control, the set of primers described by Kosaki et al. (1997) methylated and nonmethylated alleles. The reaction was hot started at 95°C for 5 min, and MS‐PCR conditions for all of the reactions were as follows: denaturation at 95°C for 15 s, annealing at 60°C for 30 s and extension at 72°C for 15 s, for 40 cycles, and final 5 min extension at 72°C. In each set of methylation‐specific PCR reactions, four samples were included. Healthy control, PWS and AS Bisulfite‐Converted genomic DNAs (from 2.4) with an input of 10 ng/μl and Negative Control (Nuclease‐free Water). The MS‐PCR products were analyzed on a 2% agarose gel with ethidium bromide (10 mg/ml, Invitrogen, USA) by horizontal electrophoresis.

### 2.5 Methylation‐sensitive high‐resolution melting

Methylation‐Sensitive High‐Resolution Melting (MS‐HRM) was performed on the 7500 Fast Real‐Time PCR System Mix (ThermoFisher Scientific, USA). Each sample was analyzed in triplicate for MS‐HRM. Primers were designed according to the principles outlined by Wojdacz and Hansen (2006). The primers used to amplify bisulfite‐treated DNA, and unmodified genomic DNA was PWS_F and PWS_R. The PCR reaction was performed in 200 μl PCR tubes with a final volume of 10 μl, containing 200 nmol/l of each primer, 5 μl of HRM‐Master Mix (ThermoFisher Scientific, USA) and 10 ng of bisulfite‐treated DNA assessed with NanoDrop Spectrophotometer. The initial denaturation (95°C, 15 min) was followed by 40 cycles for MS‐HRM of 15 s at 95°C, 1 min at 60°C and a HRM step from 60°C to 90°C rising at 0.2°C per second, and holding for 1 s after each stepwise increment. The annealing temperature of 78°C–83°C was chosen as it gave a near‐proportional amplification of methylated and unmethylated templates.

### 2.6 Bisulfite genomic (Sanger) sequencing

The DNA amplified by MS‐HRM was submitted to a DNA purification methodology, following the protocol from PureLink Quick Gel Extraction kit and PCR Purification Combo Kit (Invitrogen, USA). The purified DNA amplificons were submitted to a Bisulfite Sequencing. BigDye V. 3.1 (ThermoScientific) was used for Bisulfite genomic Sanger sequencing procedure on an ABI 3730 capillary sequencer (ThermoScientific). Sequences were compared to the reference genome (Genbank accession number: NG_01295) using Blast tools to assess the sequence identity. The software M‐Coffee tool was used to confirm the methylated and unmethylated cytosines present in the bisulfite‐treated DNA sequence.

## 3 RESULTS

**Figure 1:**
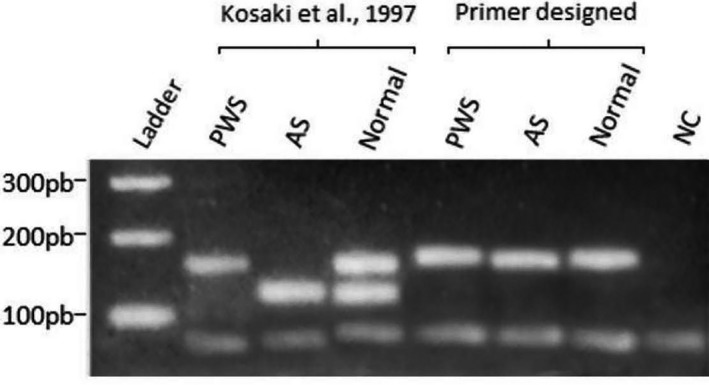
Visualization through agarose gel electrophoresis of the fragments amplified with the different pairs of primers used. The two pairs of primers described by Kosaki et al. (1997) amplify both methylated and nonmethylated allele‐producing bands with 174 bp and 131 bp respectively, being possible to distinguish abnormal methylation patterns. The unique pair of primer designed and proposed by this study amplifies both alleles equally generating a single fragment band with 196 bp. The single band of 196 bp is due to the designed primer characteristic of annealing outside of the CpG island in SNURF‐SNRPN locus. The allelic discrimination using the designed primer proposed by our study is possible through techniques such as HRM

The validation process of the MS‐HRM methodology started with an analysis of methylation pattern using the MS‐PCR technique in 43 patients suspected for PWS. The MS‐PCR amplification was performed with the two pairs of primers described by Kosaki et al. (1997). Genomic samples from PWS patients amplify only maternal allele presenting a fragmented band in agarose gel with 174 bp, the pair of primers related to the paternal allele was not possible to anneal and consequently did not amplify the DNA from paternal unmethylated allele. AS individuals present only one band with 131 bp related to the paternal allele, the maternal allele not amplified due to DNA abnormalities in maternal allele making it impossible to anneal the primers. Normal individuals showed the amplification of both alleles due to the normality of the DNA, being capable to each of two pair of primers to anneal and amplify, generating two PCR products with 174 bp (Maternal allele) and 131 bp (Paternal allele). The MS‐PCR was also performed with the unique pair of primers proposed by this study. Kosaki primers are specific for each allele (methylated maternal and unmethylated paternal) amplifying each one independently. On the other hand, the primers designed by this study do not require allelic specificity in the *SNURF‐SNPRN locus*. The consequence of that is to generate a unique PCR product for both alleles independently if the maternal or paternal allele is committed with methylation abnormalities. Individuals with PWS, AS and Normal amplified with the pair of primers proposed by this study showed a unique fragment size with 196 bp (Figure 1).

The analysis of MS‐PCR with two pairs of primers described by Kosaki et al. (1997) (methylated and unmethylated) was performed through Electrophoresis gel technique. The agarose gel technique identified 22 (51%) among 43 suspected patients with alteration in the methylation pattern at the region related to PWS or AS in chromosome 15. From these 22 patients, the agarose electrophoresis technique revealed 19 (44%) patients from the 43 studied with alterations in the paternal allele at the chromosome 15 due to the absence of the band of 131 bp, confirming the diagnosis for PWS. Furthermore, the electrophoresis gel technique detected three (6%) patients from these 22 with alteration in the maternal allele at chromosome 15 due to the absence of the band of 174 bp, compatible with the diagnosis of AS. From the 43 suspected patients studied investigated, 21 (49%) were considered normal through methylation analysis of the region revealing the presence of two bands related to the maternal and paternal alleles.

**Figure 2:**
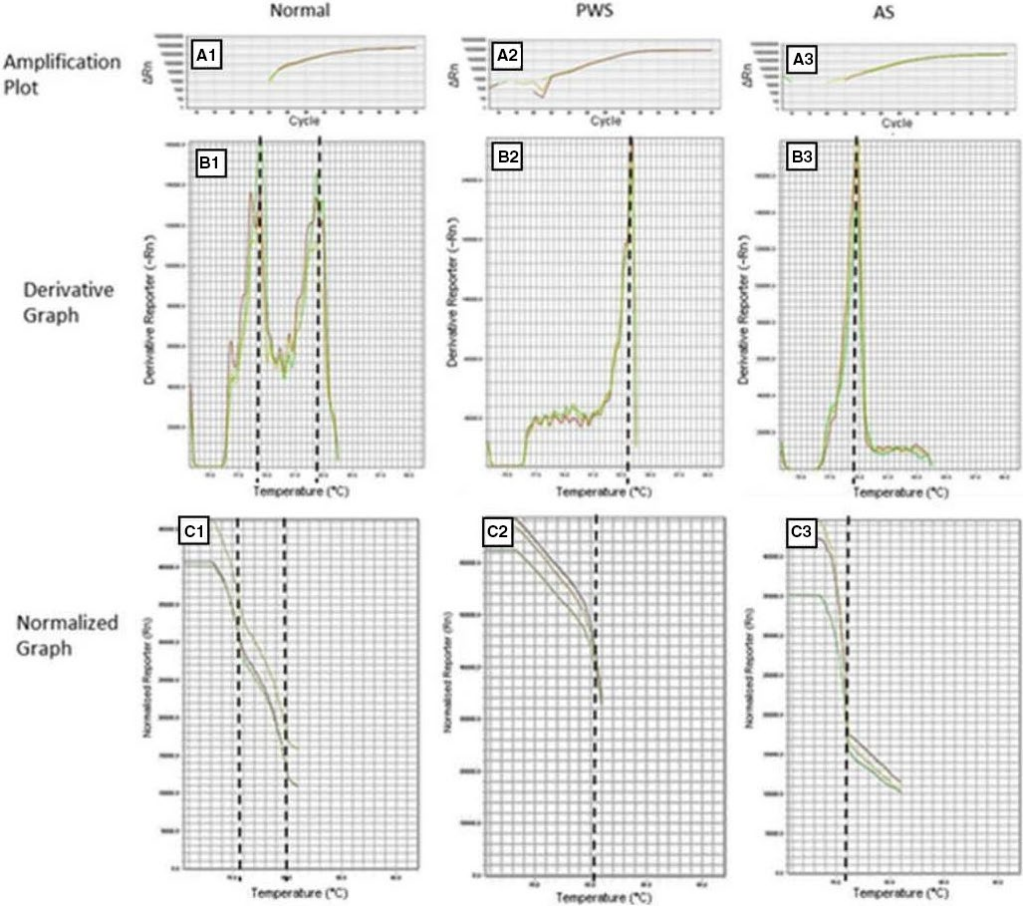
Methylation Pattern of three individuals analyzed by MS‐HRM with a unique pair of primers. Amplifications plot related to Normal (A1), PWS (A2), and AS (A3). Derivative Graphs show the melting peak to each allele. The normal patient in derivative graphs (B1) present two peaks corresponding to the paternal unmethylated and maternal methylated alleles. Normalized Graph displays the initial fluorescence issued when all products are double‐stranded and the maximum amount of dye is bound. Normal patients present to fluorescence drops corresponding to paternal and maternal allele (C1). As the temperature increases, the PCR products dissociate and the dye is released decreasing the fluorescent signal. The temperature differences between paternal and maternal allele are due to the CpG bound chemistry. Methylated cytosines are nonreactive to bisulfite conversion while nonmethylated cytosines turn uracil. Regions rich in CpG need a higher temperature to dissociate. The temperature of melting detected for maternal methylated allele was 83.3°C and 78.8°C for the paternal nonmethylated allele. The absence of paternal allele confirms a PWS (B2 and C2) while the absence of maternal allele confirms AS (B3 and C3)

The MS‐HRM technique was performed with the primers designed for the study (PWS_F/PWS_R). The amplicons were differentiated by the temperature required for dissociation of the DNA double‐strand (78°C for paternal and 83° for maternal alleles), identifying 21 (49%) patients from the 43 studied with normal methylation profile due to the presence of two peaks related to the maternal and paternal alleles. In the remaining patients, MS‐HRM detected 22 (51%) patients with alterations in the region of chromosome 15, 19 (44%) with the absence of the paternal dissociation peak characterizing PWS, and 3 (7%) with the absence of the maternal dissociation peak, confirming the diagnosis of AS (Figure 2).

The Bisulfite Sanger sequencing method confirmed that the amplicons were in the *SNURF‐SNRPN* regions. Furthermore, the M‐Coffee software compared methylated and unmethylated allele presented in *SNURF‐SNRPN* region against wild‐type DNA without bisulfite treatment (Supporting Information Figure S1) indicating all methylated cytosines position in *SNURF‐SNRPN* region. The karyotype analysis was performed in 30 (70%) of the 43 suspected patients, identifying two (7%) patients with deletions bigger than 6 Mb of DNA in the chromosome 15 at the region associated with Prader‐Willi syndrome. The GTG technique also identified 28 (93%) individuals from the study with no deletions in the same region analyzed.

We also performed a cytogenetic study through a FISH technique using two region‐specific probes (SNRPN and 15qter). The technique was performed in 19 (44%) of the 43 suspected cases, detecting four (21%) cases of deletions at chromosome 15. Furthermore, FISH analysis has confirmed GTG results in 15 (79%) of the suspects with no deletions at chromosome 15 (Supporting Information Figure S2).

The ratio of positives and negatives for the MS‐PCR, MS‐HRM, FISH, and GTG techniques as well as the proportion performed in each patient present in the study are described in Table 1.

**Table 1:** Results obtained for each technique used along the study

	MS‐PCR	MS‐HRM	FISH	GTG
PWS+AS	22 (PWS = 19)(AS = 3)	22 (PWS = 19)(AS = 3)	4 (PWS = 4)(AS = 0)	2 (PWS = 2)(AS = 0)
Normal	21	21	15	28
Not Done	0	0	24	13
Total	43	43	43	43

## 4 DISCUSSION

In newborns and young children, PWS is challenging to diagnose only by clinical examination. Molecular analysis is required for fast and precise diagnosis. Many molecular strategies evaluate the *SNURF‐SNRPN* methylation status in PWS/AS are available (Southern blotting, MS‐PCR, postrestriction PCR of bisulfite‐treated DNA, and methylation‐specific multiplex ligation‐dependent probe amplification). All the listed techniques are laborious, expensive, and time‐consuming to obtain the final result/diagnosis.

The most commonly used laboratory test for the diagnosis of PWS is based on the assessment of the absence of the paternal allele, typically using a methylation analysis approach. Methylation analysis is a sensible approach for PWS and AS diagnosis. MS‐PCR uses DNA modified by sodium bisulfite, which converts unmethylated cytosines to uracil but methylated cytosine remains nonreactive, and a set of two primers to amplify both methylated and nonmethylated allele are needed (Kosaki et al., 1997). The analysis of DNA methylation in the promoter region of the *SNURF‐SNRPN* locus will confirm the diagnosis; however, without specifying the etiology. For elucidating the genetic mechanism related to the development disease, complementary techniques such as Multiplex Ligation Probe dependent Amplification (MLPA), Fluorescence In Situ Hybridization (FISH), or Microsatellite Analysis may be used (Ramsden et al., 2010).

As mentioned by Smith and Hung (Smith & Hung, 2017), there are some issues that must be considered with regard to clinical and laboratory diagnosis: (I) Separate the few PWS patients from the vast majority of laboratory references that fall into the differential diagnosis at birth and thereafter (Dulka, Choudhary, Methratta, & Fortuna, 2013); (II) When PWS is diagnosed, it is necessary to separate individuals at risk for high recurrence (IC deletion) from those with low risk of recurrence (deletions and UPD); (III) To make the test as user‐friendly as possible—it is difficult to get blood or other tissue from the patient, and the family is not always available; and (IV) Beware of the impact of the cost of the exam in the public health system.

Our study showed a powerful approach to diagnose PWS/AS with the MS‐PCR with MS‐HRM improving and simplifying some molecular analysis steps; decreasing the procedure; avoiding the possibility of cross‐contamination among samples; quick and more straightforward interpretation results. All the bisulfite‐converted DNA from all 43 patients of the study were submitted to an MS‐PCR analysis using two pairs of primers as described by Kosaki et al. (1997); and, a single pair of primers designed and proposed by this study, followed by an electrophoresis gel technique. The MS‐PCR with two pairs of primers detected 19 patients with a single band of 174 bp suggesting the absence of the paternal allele, representing PWS. Furthermore, the technique also detected three patients with a single band of 131 bp implying the absence of the maternal allele, representing AS. The other 21 patients presented two bands of 131 bp and 174 bp, confirming they were typical for PWS/AS region. The MS‐PCR performed with a single pair of primers showed a presence of a single band due to their annealing properties is not allelic‐specific, generating similar sized products. The allelic discrimination occurred after the amplification through the High‐Resolution Melting (HRM).

After the qPCR amplification, the HRM procedure started increasing the temperature until the amplicons of the reaction completely dissociate the double‐strand DNA, allowing to detect distinct samples due to the different temperature required to the dissociation of each type of DNA. The bisulfite conversion transforms unmethylated cytosine in uracil, but methylated cytosine remains nonreactive to the conversion. Thus, methylated cytosine located within CpG island remains as cytosine and needs three hydrogen bonds to pair with guanine. Because of the difference between the chemistry, the temperature needed to dissociate double‐stranded DNA with GC‐rich content is higher than other nucleotides. Besides that, the primers proposed by this study also work as a positive control for the bisulfite conversion due to the particularity of amplifying only the converted DNA by bisulfite. A comparative analysis was performed between MS‐PCR and MS‐HRM data. The established detection ratio reached 100% agreement between both techniques detecting 19 PWS, 3 AS, and 21 normal.

Chromosomal analysis by GTG was performed in 30 patients. The technique identified 2 (7%) PWS cases caused by deletions while 28 (93%) negative karyotypes were found. A cytogenetic study was also performed through FISH technique. The FISH analysis was performed in 19 patients identifying four (21%) cases with deletions in the PWS/AS region; the remaining 15 (79%) cases of the suspects had no deletions in the region. The resolution of the FISH technique is higher than GTG analysis due to the hybridization of fluorescently labeled specific DNA sequence probes identifying with greater precision deletions and others chromosomal abnormalities.

The detection rate of deleterious changes between the FISH and GTG techniques indicated an agreement of 50%. The GTG identified two cases of deletions while the FISH technique confirmed the two cases and identified two further cases, totaling four patients. The use of probes to identify deletions proved to be efficient due to the specificity of the reaction, culminating in an increase in sensitivity and chromosomal abnormalities detection.

The gender distribution in the group of patients with clinical suspicion of PWS was 23 (53%) male and 20 (47%) female. The prevalence of detection of methylation alterations among the 22 individuals with confirmed alteration was 13 (56%) in the male group and 9 (44%) in the female group. Although the syndromes affect both sexes equally, it was possible to notice a higher prevalence of genetic alterations in the male group.

There are several methods developed for methylation analysis (Kurdyukov & Bullock, 2016); however, only a few protocols have been widely used. The bisulfite genomic sequencing can be considered as gold standard technique (Clark, Harrison, Paul, & Frommer, 1994) due to the strength in providing more detailed information about the chromosomal profile even though sensitivity is relativity low (nearly 20%) and generally inadequate for screening because it is costly and depends on the availability of equipment. The most commonly used method is methylation‐specific PCR (MS‐PCR), which uses specific primers for bisulfite‐modified and methylated DNA (Herman, Graff, Myöhänen, Nelkin, & Baylin, 1996) for example as performed by Dos Santos et al. (2016), for PWS and AS diagnosis. Despite its widespread use, MS‐PCR has several significant limitations (Cottrell & Laird, 2003). As with other techniques that use the combination of PCR primers to increase specificity, a competition of primers pairs may occur, leading to false positives if the primers are poorly designed or used at low temperatures. Our study designed only one pair of primers, avoiding the competition for reaction reagents and reducing the risk of primer dimer formation. Besides that, sometimes it is difficult to avoid CpG dinucleotides in the primers to amplify CpG islands, it has been shown that some CpGs are required in the primer sequence; otherwise, the PCR bias may lead to a significant underestimate of the degree of methylation (Wojdacz & Hansen, 2006). Accordingly, we adopted the strategy of using primers containing limited numbers of CpG and manipulating the hybridization temperature to control the trend of PCR amplification in the design MS‐HRM assays. Procter, Chou, Tang, Jama, and Mao (2006), described a molecular technique based on bisulfite conversion, using SYBR Green, which is inappropriate for diagnosis due to the characteristic of being a nonsaturating intercalating dye. The HRM analysis is dependent on the use of high sensitivity fluorescence detection instrumentation with the use of saturating intercalating dyes and software, allowing the analysis of the fusion profiles of the PCR products. We developed the HRM for the discrimination between methylated and unmethylated sequences after modification of the target DNA by bisulfite. In this context, we tested the pairs of primers with two successful HRM kits with success in both reagents, which can be used on different platforms and different commercial kits.

The amplicon size directly affects the sensitivity of genotyping. Shorter amplicon fragments generally allow better discrimination of small sequence differences. As the size of the amplicon decrease, the differences in melting temperature between the genotypes increase, allowing a better differentiation between normal and altered samples. Chou, Lyon, and Wittwer (2005) described a 100‐300 bp amplicon length as a recommended size for HRM analysis. In this regard, the fragment described here presents 196 bp, lower when compared to Procter et al. (2006) (322 bp) and White, Hall, and Cross (2007) (238 bp). The fragment size is directly related to HRM accuracy, shorter amplicons offer an ease results interpretation without risk of overlapping peaks since the melting temperature of the maternal (methylated), and paternal (unmethylated) alleles are 83°C and 78°C, respectively.

MS‐HRM can provide the possibility to find changes in the genome by differences in the curvature of the dissociation curve, indicating changes in the gene sequence or the methylation pattern without the need for sequencing. It is essential for the differential diagnosis of patients presenting complex clinical phenotypes, including developmental delay and intellectual disability. MS‐HRM approaches can be used to accurately identify patients with these conditions, assisting in the interpretation of genetic variations of unknown significance in these conditions. Finally, it can be used as part of the molecular routine screening protocols in patients with a wide range of developmental delay and intellectual disability disorders, unsolved cases or those requiring differential diagnosis.

Changes in the expression of imprinted genes have been associated with an increasing number of human diseases, including PWS and AS. Besides, the methylation profile may alter the development of different cell types through a pleiotropic effect on the genome. Witoelar et al. (1997) observed a shared status of the methylation profile and the association between Parkinson's disease and autoimmune diseases. Changes in the methylation profile such as DNA hypermethylation may reveal potential new biomarkers for prognostic evaluation in different types of cancer (Kristensen & Hansen, 2009; López et al., 2017). The methylation of genomic DNA in the CpG islands is a mechanism of gene expression control. Quick detection of changes in profile methylation as observed with the methodology MS‐HRM allows clarifying diagnosis and understanding of new mechanisms identification of genetic processes associated with particular phenotypes.

The MS‐HRM technique offers quick, high precision, and sufficient robustness for clinical use with the implementation of a multiplexed protocols for DNA methylation to improve efficiency. Due the analysis occurs without the need for additional techniques, the MS‐HRM methodology provides a reduced risk of cross‐contamination between samples which is a significant problem in both research and diagnostic laboratories.

The method herein presented has significant advantages over conventional analysis using MS‐PCR followed by Electrophoresis gel. The MS‐HRM with a unique pair of primers does not need additional techniques such as agarose gel electrophoresis, reducing the time taken to obtain an accurate result and reducing the risks of cross‐contamination between samples. Due to the high capacity of parallel samples analyses, the method also shows to be robust and with a fast and straightforward interpretation of the results providing a quick test result. The sensitivity of MS‐HRM allows the detection of a tiny fraction of sample is important in the analysis of samples, which are difficult to obtain especially from newborn hypotonic infants. Besides, high reproducibility of HRM makes this method suitable for both research and diagnostic applications in the study of PWS and AS syndromes. A quick and accurate diagnosis of PWS or AS allows effective medicament intervention, avoiding several characteristics of these disorders, providing a better quality of life.

## CONFLICT OF INTEREST

All authors have no conflicts of interest to declare.
