# Supplementary material for: A rapid and accurate methylation‐sensitive high‐resolution melting analysis assay for the diagnosis of Prader Willi and Angelman patients
Source: Mol Genet Genomic Med. 2019 Apr 29;7(6):e637. doi: 10.1002/mgg3.637 (PMC6565559; doi:10.1002/mgg3.637)
Supplement: Supplementary file 1 [file MGG3-7-e637-s001.pdf]

Supporting information: Figure 1 S

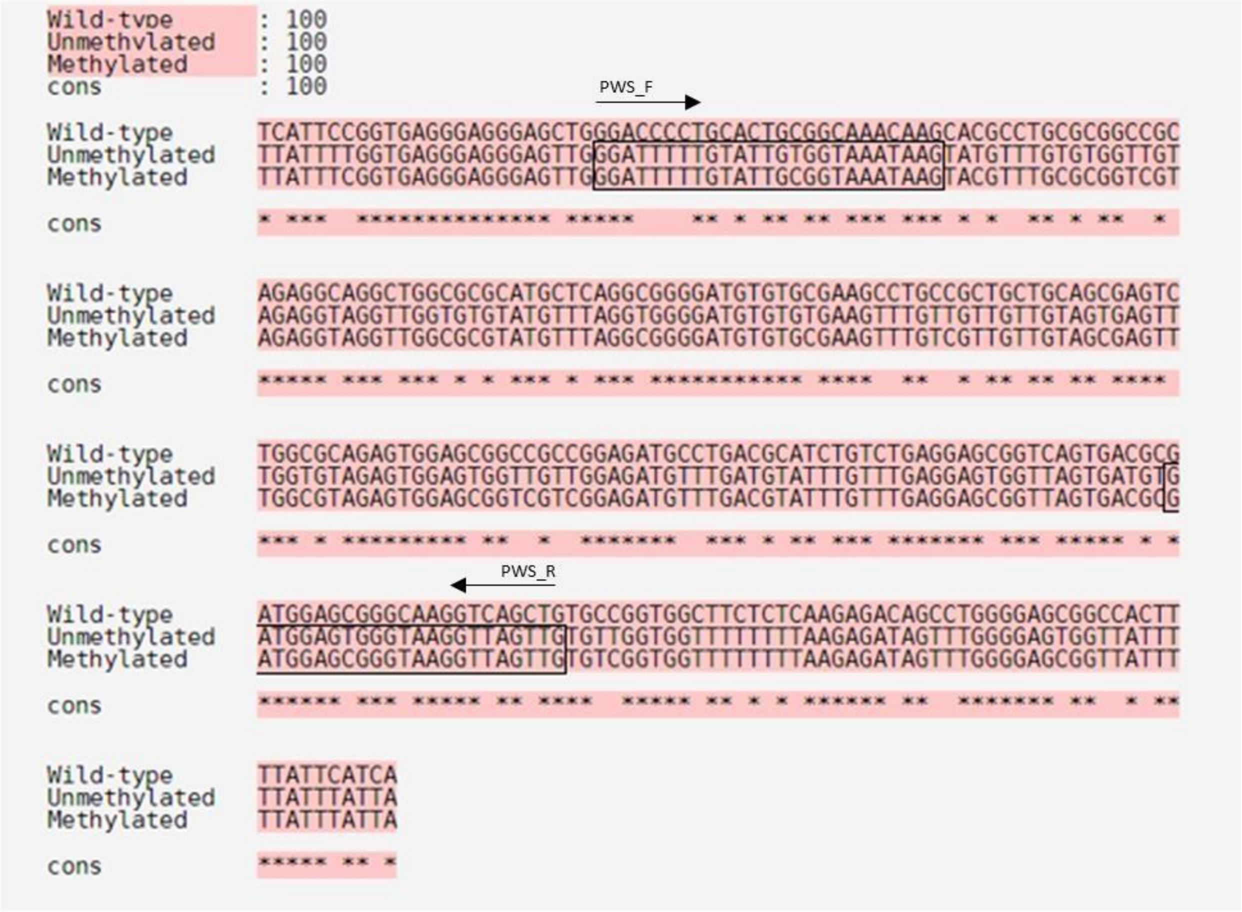

Fig. 1 S - Bisulfite treated DNA amplified with PWS_F and PWS_R primers followed by a bisulfite Sanger sequencing. The bisulfite sequence DNA was compared to a wild-type DNA to identify the nucleotides presented in Wild-type, Unmethylated and Methylated alleles. The nucleotide presented in all of 3 situations was marked with *. The variance along this sequence region is directly related to the unmethylated cytosines converted by bisulfite treatment. The arrows and boxes indicates the primer direction and the localization of each primer, respectively.
