# Supplementary material for: A rapid and accurate methylation‐sensitive high‐resolution melting analysis assay for the diagnosis of Prader Willi and Angelman patients
Source: Mol Genet Genomic Med. 2019 Apr 29;7(6):e637. doi: 10.1002/mgg3.637 (PMC6565559; doi:10.1002/mgg3.637)
Supplement: Supplementary file 2 [file MGG3-7-e637-s002.pdf]

Supporting information: Figure 2 S

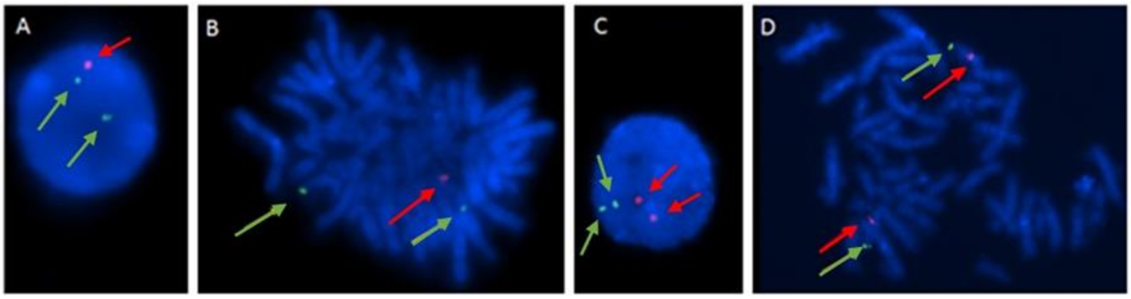

Fig. 2 S - The FISH technique in peripheral blood cells from PWS patients (*A and B*) and normal (C and D), using the SNRPN (red) and 15qter (green) probes. In A and C, *metaphase nucleus* of PWS and normal patients are shown, respectively. In B and D, metaphase spreads chromosomes of PWS and normal patients are exemplified respectively.
